# Pediatric sepsis diagnostic and prognostic biomarkers: pancreatic stone protein, copeptin, and apolipoprotein A-V

**DOI:** 10.1038/s41390-023-02499-0

**Published:** 2023-02-08

**Authors:** Nagwan Y. Saleh, Hesham M. Aboelghar, Mohamed I. Garib, Mohammed S. Rizk, Asmaa A. Mahmoud

**Affiliations:** 1 Department of Pediatrics, Faculty of Medicine, Menoufia University, Shebin Elkom, Egypt; 2 Department of Medical Biochemistry and Molecular Biology, Faculty of Medicine, Menoufia University, Shebin Elkom, Egypt

## Abstract

**Background:**

We assessed serum concentrations of pancreatic stone protein (PSP), copeptin, and apolipoprotein A-V (APOA5) biomarkers for the diagnosis and prognosis of pediatric sepsis, a condition associated with high mortality.

**Methods:**

This prospective study included 180 children admitted to the Pediatric Intensive Care Unit and 100 healthy controls at Menoufia University Hospital. Pediatric Risk of Mortality (PRISM), Pediatric Index of Mortality-2 (PIM2), and Pediatric Sequential Organ Failure Assessment (pSOFA) scores were calculated. Serum PSP, copeptin and APOA5 were measured once within 24 h of admission.

**Results:**

PSP, copeptin, and APOA5 were significantly higher in the patients than in the controls (*p* < 0.001). PSP and copeptin were increased among children who required mechanical ventilation (MV), had multiple organ dysfunctions, and were non-survivors, but APOA5 was decreased in those children. Logistic regression analyses showed that high pSOFA, high PSP and copeptin, low APOA5, and use of MV were associated with mortality. The receiver operating characteristic revealed that the area under the curve (AUC) for APOA5, copeptin, and PSP (0.965, 0.960, and 0.868, respectively) demonstrated high sensitivity (96%, 94%, and 80%) for sepsis diagnosis. The AUC values for PSP, copeptin, and APOA5 were 0.709, 0.705, and 0.571, respectively, with sensitivities of 74%, 58%, and 58% for mortality prediction.

**Conclusions:**

PSP, copeptin, and APOA5 are promising diagnostic biomarkers for pediatric sepsis but inadequate predictors of mortality.

**Impact:**

- Apolipoprotein A-V (APOA5), copeptin, and pancreatic stone protein (PSP) are acute-phase proteins with diagnostic value in evaluating critically ill pediatric patients with sepsis and detecting sepsis severity.
- PSP and copeptin had the power to discriminate non-survivors from survivors.
- APOA5 was less powerful than the other biomarkers in discriminating between survivors and non-survivors.

## Introduction

Sepsis is the leading cause of death worldwide among children under 5 years of age, accounting for 19% of all pediatric deaths. Furthermore, pediatric sepsis accounts for 8% of Pediatric Intensive Care Unit (PICU) admissions.^1^ The most prevalent comorbidities in children with sepsis were respiratory diseases (including those that require ventilators), neurologic, metabolic, and oncologic illnesses, congenital heart disease, and inherited or acquired immunodeficiency. The use of a central venous catheter is also associated with pediatric sepsis.^2^

In 2020, the first international guidelines for the Pediatric Surviving Sepsis Campaign (SSC) listed non-cardiovascular dysfunction as one definition of sepsis.^3^ Clinical symptoms of pediatric sepsis include the presence of suspected infection and abnormal physical examination findings, such as poor perfusion, hypotension, tachycardia, temperature anomalies, and altered mental status.^4^

During sepsis, the serum concentrations of some proteins, such as pancreatic stone protein (PSP), copeptin, and APOA5, have been observed to increase. PSP is a globular polypeptide containing domains of C-type lectins, calcium-dependent glycan-binding proteins that cause pro-inflammatory activity by stimulating adhesion and signaling receptors during homeostasis and activation of innate immunity.^5^ PSP levels have been observed to increase before the onset of sepsis and continue to increase during septic shock.^6^ Copeptin is the 39-amino-acid C-terminal portion of the vasopressin precursor pre-provasopressin. It is released into the portal circulation of the neurohypophysis after cleavage in the hypothalamus. Individuals with severe sepsis have more copeptin, which increases 30-fold in septic shock patients.^7^ APOA5 (apolipoprotein A-V) helps regulate triglyceride homeostasis. The acute-phase reactions in sepsis induce dyslipidemia, characterized by high triglyceride levels and low high-density lipoprotein cholesterol values.^8^ Serum APOA5 concentrations in juvenile patients with sepsis have been found to be substantially greater than in adult patients.^9^ The objective of the present study was to determine whether the levels of PSP, copeptin, and APOA5 can serve as diagnostic and prognostic sepsis biomarkers.

## Subjects and methods

### Study design

In this prospective study, we enrolled 180 critically ill children admitted to a 10-bed PICU at Menoufia University Hospital, Egypt, from March 2021 to December 2021. The Scientific and Ethical Committee approved the study protocol of Menoufia University, and informed consent was obtained from parents before enrolling their children in the study. Critically ill children in the PICU aged 1 month to 18 years were included in this study. Exclusion criteria were (1) age less than 1 month or more than 18 years and (2) any children who received corticosteroid or immunosuppression drugs and oncology patients and (3) inability to follow-up for the first 30 days after discharge. The control group was composed of 100 un-hospitalized, healthy age- and sex-matched children. All control group children were found to be healthy on examination.

### PICU patient cohort

The included patients were diagnosed according to the International Pediatric Sepsis Consensus Conference, characterizing septic, severe septic, and septic shock groups.^10^ Sepsis is a systemic response to an infectious stimulus characterized by two or more of the following, resulting in infection: (a) a temperature of more than 38 °C or less than 36 °C, (b) pulse rate >90 beats**/**min, (c) breath rate >20 breaths/min or PaCO_2_ < 32 mm Hg, and (d) white blood cell count (WBC) of >12,000/mm^3^ or <4000/mm^3^, or >10% immature (band) formation in a total blood count. Severe sepsis is defined as sepsis plus the following: cardiovascular organ dysfunction, acute respiratory distress syndrome (ARDS), or two or more other organ dysfunctions. Septic shock is a subgroup of severe sepsis diagnosed as hypotension caused by sepsis that continues despite sufficient fluid replacement.

### Study outcomes

The outcome measures were sepsis diagnosis in critically ill children, mortality during hospital admission or during the 30-day follow up period after hospital discharge, the length of stay (PICU and hospital), need and duration of mechanical ventilation (MV), use of vasopressor drugs, and sepsis severity as indicated by pediatric multiple-organ dysfunction syndrome (P-MODS) the impaired function of more than two organs (lung, heart, brain, liver, kidney, and circulatory and metabolic systems)^10^ and disseminated intravascular coagulation (DIC) scores. A DIC score ≥5 is consistent with DIC according to the International Society on Thrombosis and Hemostasis subcommittee guidelines.^11^

### Study methods

We collected the complete history of all children participating in this study, including age, sex, admission data, and length of stay in the PICU and the inpatient department. Vital signs, anthropometric measurements, and examination of all body systems were also assessed. PICU scoring systems were applied, including the (1) Pediatric Risk of Mortality Score (PRISM), (2) Pediatric Index of Mortality-2 (PIM2), and (3) Pediatric Sequential Organ Failure Assessment Scale (pSOFA). The PRISM score was calculated within 24 h of admission for each patient, using 14 clinical and laboratory variables. Values for these variables were entered into the PRISM application (http://www.sfar.org/scores2/prism2.php), which calculates the expected death rate.^12^ PIM2 is a more rapid technique for which scores are estimated within 1 h of in-person contact with the patient, and scores correspond to a predicted mortality rate.^13^ The pediatric Sequential Organ Failure Assessment Scale (pSOFA) is used to assess organ dysfunction. Depending on the patient’s baseline risk level, a pSOFA score of 2 or greater corresponds to a 2- to 25-fold greater risk of death than in patients with pSOFA scores was less than 2.^14^

Arterial blood gases, random blood glucose, and complete blood count (CBC) were analyzed (Pentra ABX 80 analyzer; Horiba, Paris, France). C-reactive protein (CRP), hepatic function (alanine aminotransferase and aspartate aminotransferase) were determined using a kinetic UV-optimized IFCC method (LTEC Kit, England). Renal function (blood urea and serum creatinine) was determined colorimetrically (Diamond Diagnostic kit, Germany). Blood culture, chest X-ray radiography, brain CT, and other laboratory or radiological analyses were performed as needed.

### The procedures

PSP, copeptin, and APOA5 serum concentrations were measured once from blood samples were withdrawn from all patients (within 24 h of PICU admission) and control group participants. All were analyzed using enzyme-linked immunosorbent assay (ELISA) kits (copeptin kit: Shanghai SunRed Biological Technology Co., Shanghai, China; PSP kit: Human PSP, China; APOA5 kit: MultiScience [Lianke] Biotech, Co., Ltd., Hangzhou, China).

### Sample size determination

A previous study^15^ reported a serum APOA5 value of 1096 ng/ml and a case fatality rate of 19.3% with a specificity of 86.67%. Based on this information, we calculated a minimum sample size of 178 diseased cases with a total sample size of 221.

### Statistical analysis

Data were statistically analyzed using SPSS (version 19, SPSS Inc, Chicago, IL). Descriptive statistics included arithmetic medians and interquartile ranges (IQRs) of quantitative data and numbers and percentages of qualitative data. Analytical statistics included the Chi-square (*χ*^2^) test, Student’s *t* test, Mann–Whitney test, and Fisher’s exact test. We used logistic regression models to determine the ability of biomarkers to predict mortality. Receiver operating characteristics (ROC) analysis was performed for the diagnostic and prognostic powers of the biomarkers, and other variables. *p* Values <0.05 were considered significant.

**Table 1:** Baseline characteristics of the patient and control groups.

Studied variables	Patients (*n* = 180)	Control (*n* = 100)	Test of significance	*p* value
Age (month)	13 (5–45)	12 (9–48)	*U* = 0.428	0.669
Male, sex	86 (48%)	53 (53.0%)	*χ*^2^ = 0.142	0.706
Weight (kg)	8.5 (5–14)	9.7 (9–17)	*U* = 0.449	0.655
Height (cm)	79 (65–95)	77 (73–101)	*T* = 0.315	0.755
BMI (kg/m^2^)	8.5 (5.5–14)	9 (6-14)	*U* = 0.449	0.686
Mechanical ventilation needed	45 (25%)	–	–	–
Mechanical ventilation duration (h)	4 (2–7.2)	–	–	–
PICU stay/day	6 (4–10)	–	–	–
Hospital stay/day	16 (9–28)	–	–	–
PRISM mortality risk %	26.8 (9–70.5)	–	–	–
PIM2 mortality risk %	7.9 (3–65)	–	–	–
pSOFA score	6 (5–9)	–	–	–
Mortality	29 (16.1%)	–	–	–
PSP (ng/ml)	33.99 (10.87–185.8)	6.78 (4.74–13.53)	*U* = 3.806	<0.001*
Copeptin (ng/ml)	3.09 (2.44–4.95)	1.04 (0.59–1.73)	*U* = 4.4.0	<0.001*
Apolipoprotein A-V (ng/ml)	882.8 (381–1600)	671 (283–769.3)	*U* = 4.65	<0.001*

Data are expressed as number (%) or median (IQR).

*U* Mann–Whitney *U*-test, *X*^2^ Chi- square, *BMI* body mass index, *PICU* Pediatric Intensive Care Unit, *PRISM* Pediatric Risk of Mortality, *PIM2* Pediatric Index of Mortality-2, *pSOFA* Pediatric Sequential Organ Failure Assessment Score, *PSP* pancreatic stone protein.

*Statistically significant.

## Results

### The basic characteristics of the studied groups are shown in Table 1

A total of 180 patients were included in our study along with 100 controls and 16.1% of the patients group was died.

### The clinical severity, laboratory analyses, and outcome data of the studied groups

Of the included PICU cohort, 95 (52.8%) had sepsis, 57 (31.7%) had severe sepsis, and 28 (15.5%) had septic shock. Twenty-nine children (16.1% of the PICU cohort) died. Sepsis originating from respiratory conditions was the most common source (54.3%). Blood cultures were positive for bacteria in 131 of 180 (72.8%) septic children, 68% of which were Gram-negative, 17% were Gram-positive, and 15% were mixed infections. Need and duration of MV, vasopressor use, P-MODS, DIC, and pSOFA scores were significantly higher (*p* = 0.001, 0.047, <0.001, <0.001, <0.001, and 0.002, respectively) in the septic shock group than the septic and severe septic groups. Moreover, PRISM, PIM2 mortality risk %, and mortality rate were also significantly greater (*p* < 0.001, <0.001, and 0.005, respectively) in the septic shock group than the other septic groups, as were serum levels of creatinine and CRP (*p* = 0.048 and 0.019) (Table 2).

**Table 2:** Clinical severity, laboratory, and outcome data of the studied groups.

Studied variables	Sepsis (*n* = 95)	Severe sepsis (*n* = 57)	Septic shock (*n* = 28)	*p* value
Age (month)	12 (4.2–44.2)	20 (11.3–42)	24 (3–120)	0.783
Male, sex	38 (40%)	25 (43.8%)	12 (42.8%)	0.712
Clinical severity				
MV needed	9 (9.5%)	15 (26.3%)	21 (75.0%)	0.001*
MV duration (h)	96 (40–158)	120 (48–169)	144 (59–180)	0.047*
Use of vasopressor	10 (10.5%)	15 (26.3%)	28 (100%)	<0.001*
P-MODS	0 (0–1)	2 (0–3)	7 (3–9)	<0.001*
DIC score	2 (0–2)	3 (1–3)	5 (3–5)	<0.001*
pSOFA	5 (4–7)	8.5 (7–10.2)	9 (7–12)	0.002*
Laboratory data				
WBCs (1000/µl)	14.8 (8.6–19.9)	18.7 (8.3–21.2)	17.8 (4.3–20.9)	0.909
Platelets (1000/µl)	315 (256–402)	237 (190–540)	219 (118–263)	0.968
Creatinine (mg/dl)	0.6 (0.3–0.9)	0.55 (0.57–1.8)	0.90 (0.60–2.1)	0.048*
CRP (mg/l)	32 (12–48)	34 (17.5–48)	48 (26–84)	0.019*
PSP (ng/ml)	19.7 (10.2–49.2)	68.4 (55.2–100.6)	143.0 (83.6–230.1)	<0.001*
Copeptin (ng/ml)	2.57 (2.33–3.09)	5.08 (4.60–5.65)	6.57 (6.50–7.31)	<0.001*
Apolipoprotein A-V (ng/ml)	1500 (1500–1600)	1154.5 (861.5–1500)	842.1 (505.8–951.6)	<0.001*
Outcome				
PICU stay/day	6.5 (5–10)	8.5 (9–16)	8 (5–17)	0.148
Hospital stay/day	15 (10–25)	16 (12–31)	17 (13–29)	0.552
PRISM mortality risk %	9 (9–15)	26.8 (19–36.5)	45.5 (39–70.5)	<0.001*
PIM2 mortality risk %	6.9 (4.5–11.6)	17.5 (4.19–23.03)	42.6 (5.21–65)	<0.001*
Mortality	5 (5.3%)	9 (15.8%)	15 (53.6%)	0.005*

Data are expressed as number (%) or median (IQR).

*MV* mechanical ventilation, *P-MODS* Pediatric-Multiple Organ Dysfunction, *DIC* disseminated intravascular coagulation, *pSOFA* Pediatric Sequential Organ Failure Assessment Score, *WBC* white blood cells, *CRP* C-reactive protein, *PSP* pancreatic stone protein, *PICU* Pediatric Intensive Care Unit, *PRISM* Pediatric Risk of Mortality, *PIM2* Pediatric Index of Mortality-2, *PSP* pancreatic stone protein.

*Statistically significant.

### Serum biomarker measurements

Serum levels of PSP, copeptin, and APOA5 were significantly greater in the PICU patients than in the control group (*p* < 0.001). Serum levels of PSP and copeptin were significantly elevated in the PICU patient with higher levels in the septic shock group (*p* ≤ 0.001 and 0.015, respectively). Serum levels of APOA5 were significantly decreased in the PICU patient with a lower level in the septic shock group (*p* ≤ 0.001) (Table 2).

**Fig. 1:**
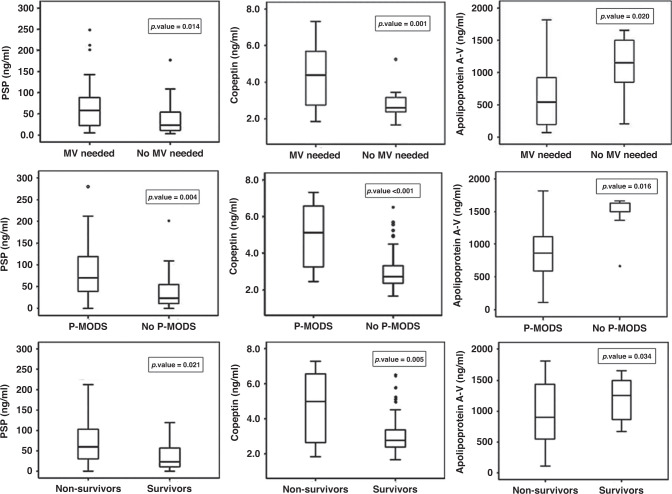
Comparison of pancreatic stone protein (PSP), copeptin, and apolipoprotein A-V among different patient subgroups. (1) PSP, copeptin and apolipoprotein A-V in MV needed and No MV needed. (2) PSP, copeptin and apolipoprotein A-V in P-MODS and in No P-MODS. (3) PSP, copeptin and apolipoprotein A-V in Non-survivors and Survivors.

### Comparison of PSP, copeptin, and APOA5 among different patient subgroups is shown in Fig. 1

PSP and copeptin were significantly higher in patients who required MV than those who did not (*p* = 0.014 and 0.001, respectively), but APOA5 was significantly lower in patients who required MV (*p* = 0.020). Patients with P-MODS had higher levels of PSP and copeptin and a lower level of APOA5 (*p* = 0.004, <0.001, and 0.016, respectively). PSP and copeptin were significantly higher in non-survivors than in survivors (*p* = 0.021, 0.005). The opposite was found for APOA5 (*p* = 0.034).

**Table 3:** Univariate and multivariate logistic regression analyses for prediction of mortality by different variables.

Variable	Odds ratio (95% CI)	*p* value
Univariate logistic regression analysis		
pSOFA	4.645 (2.516–10.805)	<0.001*
Mechanical ventilation need	4.625 (2.561–10.308)	<0.001*
PRISM risk mortality %	1.832 (1.740–2.939)	0.002*
PIM risk mortality %	1.342 (1.903–2.984)	0.007*
P-MODS	7.621 (2.343–25.231)	**0.001**
PSP (ng/ml)	1.852 (1.730–1.945)	0.003*
Copeptin (ng/ml)	1.625 (1.093–2.416)	0.016*
Apolipoprotein A-V (ng/ml)	0.799 (0.732–0.999)	0.017*
Multivariate logistic regression analysis		
pSOFA	4.704 (2.496–10.999)	0.050*
Mechanical ventilation need	12.121 (1.871–14.326)	0.049*
PRISM risk mortality %	1.807 (1.626–2.04)	0.099
PIM risk mortality %	1.09 (1.053–2.194)	0.350
P-MODS	2.289 (2.262–19.3)	0.459
PSP (ng/ml)	1.822 (1.760–1.967)	0.002*
Copeptin (ng/ml)	1.680 (1.031–2.782)	0.036*
Apolipoprotein A-V (ng/ml)	0.755 (0.730–0.998)	0.016*

*pSOFA* Pediatric Sequential Organ Failure Assessment score, *PRISM* Pediatric Risk of Mortality, *PIM2* Pediatric Index of Mortality-2, *P-MODS* Pediatric-Multiple Organ Dysfunction, *PSP* pancreatic stone protein, *OR (95% CI)* odds ratio and 95% confidence interval.

*Significant.

Bold values mean statistically significant.

### Univariate and multivariate logistic regression analyses for the prediction of mortality by different variables are shown in Table 3

Univariate logistic regression analysis showed that MV required, higher pSOFA, PRISM, PIM2, and P-MODS scores, increased levels of PSP and copeptin and decreased level of APOA5 were significantly associated with mortality. Multivariate logistic regression analysis showed that MV requirement, higher pSOFA, and higher PSP and copeptin levels and lower APOA5 levels were the most significant independent risk factors for mortality.

### ROC curve analysis

**Fig. 2:**
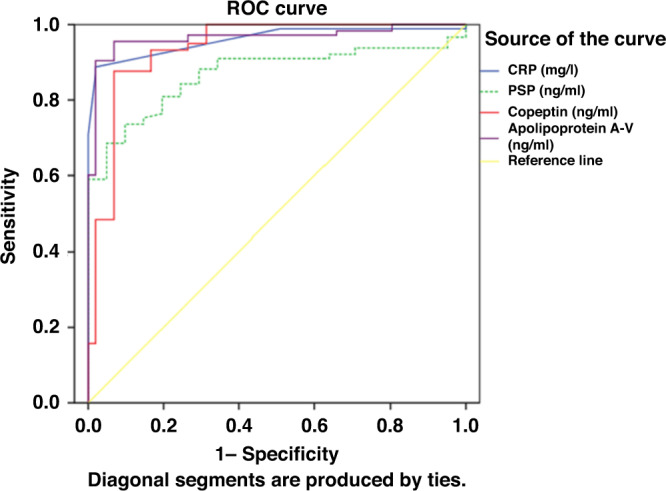
ROC curve of PSP, copeptin, ApoA5, and CRP for sepsis diagnosis.

**Fig. 3:**
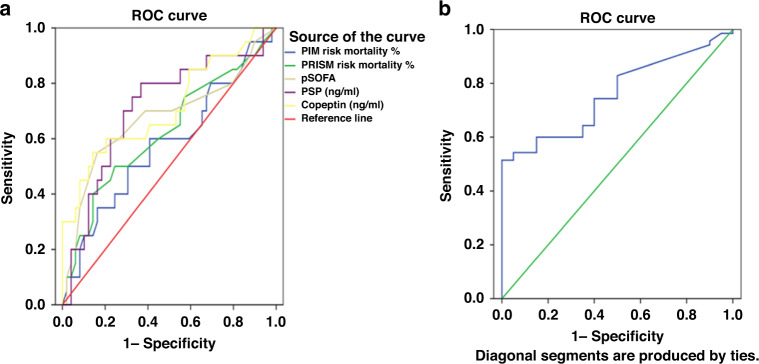
ROC curve of biomarkers and mortality scores for mortality prediction. **a** ROC curve of PSP, copeptin, pSOFA, PRISM, and PIM for mortality prediction. **b** ROC curve of ApoA5 for mortality prediction.

ROC curve analysis showed that APOA5, CRP, copeptin, and PSP predicted pediatric sepsis with high sensitivity. APOA5 level was the best predictor, with an AUC of 0.965, and demonstrated 96% sensitivity and 90% specificity at a cut-off level of >250 ng/ml. The AUC of copeptin, CRP and PSP were 0.960, 0.917, and 0.868, respectively (Table 4 and Fig. 2). PSP and pSOFA were the most significant discriminators for mortality, and PSP levels and pSOFA scores both had an AUC of 0.709 for mortality prediction, followed by copeptin (AUC = 0.705). APOA5 level, and PRISM and PIM2 scores were poorer discriminators for predicting mortality (Table 4 and Fig. 3a, b).

**Table 4:** Validity of serum biomarkers and clinical variables to predict sepsis and mortality.

Variable	AUC (95% CI)	*p* value	Cut-off level	Sensitivity (%)	Specificity (%)
Validity of serum biomarkers and clinical variables to predict sepsis					
CRP (mg/l)	0.917 (0.904–0.970)	0.001*	>11	87%	100%
PSP (ng/ml)	0.868 (0.826–0.911)	0.001*	>9.79	80%	75%
Copeptin (ng/ml)	0.960 (0.938–0.982)	0.001*	>1.81	94%	77%
Apolipoprotein A-V (ng/ml)	0.965 (0.944–0.987)	0.001*	>250	96%	90%
Validity of serum biomarkers and clinical variables to predict mortality					
PRISM risk mortality %	0.611 (0.453–0.769)	0.159	>8.5	58%	59%
PIM risk mortality %	0.573 (0.412–0.734)	0.351	>9.8	42%	72%
pSOFA	0.709 (0.558–0.861)	0.008*	>6.50	74%	62%
PSP (ng/ml)	0.709 (0.564–0.854)	0.008*	>54	74%	72%
Copeptin (ng/ml)	0.705 (0.557–0.854)	0.009*	>3.63	58%	76%
Apolipoprotein A-V (ng/ml)	0. 571 (0.424–0.718)	0.365	<951	58%	53%

*CRP* C-reactive protein, *PSP* pancreatic stone protein, *PRISM* Pediatric Risk of Mortality, *PIM2* Pediatric Index of Mortality-2, *pSOFA* Pediatric Sequential Organ Failure Assessment score, *AUC (95% CI)* area under the receiver operating characteristic curve and 95% confidence interval.

*Significant.

## Discussion

The three biomarkers assessed in this study for their value in the diagnosis and prognosis of pediatric sepsis act differently in the body. PSP activates neutrophil granulocytes and is released mainly from the pancreas in the early stage of sepsis. The endogenous stress of the patient, reflecting sepsis severity, was determined by measuring copeptin. APOA5 was used as a biomarker for sepsis-induced dyslipidemia.

Serum levels of PSP, copeptin, and APOA5 were significantly higher in the pediatric sepsis group than in the control group. The AUC values of them suggest that these biomarkers could be used as acute phase reactants and could have a critical role in sepsis diagnosis.

Prediction of disease severity and mortality is vital in pediatric critical units. Our findings showed that PSP and copeptin levels in the blood were substantially greater in the septic shock group than in the other sepsis groups. In contrast to PSP and copeptin, serum APOA5 levels were significantly lower in the septic shock group and non-survivors. Our findings support using these biomarkers in the assessment of pediatric sepsis severity.

A considerable increase in copeptin levels was previously found in patients with sepsis and septic shock, with non-survivors having higher levels than survivors.^16^ Serum copeptin was also found to be higher in critically ill patients with more than one organ failure.^17^ Jiang et al.^18^ reported higher copeptin levels in non-surviving sepsis patients. Previous research also found a significant positive correlation between copeptin serum levels and APACHE II disease severity scores.^18^ However, these results are not consistent as other researchers found no significant difference in copeptin levels between patients with sepsis or severe sepsis and the healthy controls.^19^

In a previous study, pediatric patients with sepsis admitted to the PICU had significantly higher APOA5 levels than healthy children and significantly lower levels in non-survivors than in survivors. Patients with MODS and shock had decreased serum levels of APOA5.^15^

PSP serum levels were previously found to increase 5 days^20^ and 3 days^21^ before sepsis diagnosis. PSP levels were higher in burn patients before they developed sepsis. Furthermore, septic shock is associated with a significant increase in PSP levels.^6^ In severely ill adult sepsis patients, baseline PSP levels were found to be increased, with non-survivors having higher serum PSP than survivors.^22^ Non-surviving PICU patients were also found to have higher PSP concentrations than survivors in a pediatric cohort in which 37% of PICU patients died.^23,24^ Significant positive correlations between PSP and mortality were observed in patients with or without sepsis and between PSP levels and WBC counts.^25^ Schlapbach et al.^26^ testing PSP for the diagnosis of early-onset sepsis.

The most common origin of sepsis in our PICU cohort was the respiratory system (54.3%), and sepsis was associated with infections of Gram-negative (68%), Gram-positive (17%), and mixed (15%) microflora. In a previous study, the most prevalent infected site in 214 pediatric sepsis patients was the respiratory tract (40.2%), of which Gram-positive infections accounted for 51.4%, Gram-negative infections 35.5%, fungal infections 9.8%, and mixed infections 3.3%.^23^

The highest independent mortality risk factors in our patients as determined by logistic multivariate regression analyses were higher pSOFA, MV requirement, and high levels of PSP and copeptin and low levels of APOA5. Our results are consistent with a previous study^27^ in which the univariate logistic regression analysis of variables revealed that increased mortality in PICU patients with sepsis was significantly associated with an increase in pSOFA score (>4) and MV use. Multivariate regression analysis of an increase in pSOFA score (>4) was significantly associated with increased mortality, with a calculated odds ratio of 3.6 (95% CI, 1.30–9.93).

In a previous PICU study, univariate logistic regression analysis showed that a low APOA5 level was an independent risk factor for PICU mortality in pediatric patients with sepsis.^15^ Furthermore, multivariate logistic regression analysis in another PICU study revealed that PSP was an independent factor in the prognosis of sepsis in pediatric patients.^23^ Elevated copeptin levels in patients were also associated with an increased risk of mortality^16^ and were predictive of mortality in critically ill patients with sepsis.^28^

The ROC assessment of the diagnostic power of the biomarkers and CRP showed that APOA5, copeptin and CRP levels had excellent AUC values (0.965, 0.960, and 0.917, respectively) for sepsis diagnosis. For APOA5, the cut-off serum concentration was 250 ng/ml with 96% sensitivity and 90% specificity. The copeptin cut-off concentration was 1.81 ng/ml with a sensitivity of 94% and a specificity of 77%, and for CRP, the cut-off concentration was 11 mg/l with 87% sensitivity and 100% specificity. PSP concentration AUC values were 0.868 for the detection of sepsis with a cut-off level of 9.79 ng/ml with 80% sensitivity and 75% specificity.

The AUC of copeptin has previously been found to be 0.845 for predicting sepsis or septic shock with a cut-off of 23.2 pmol/l, with 74% sensitivity and 87% specificity.^29^ Serum APOA5 level exhibited an AUC of 0.753 (95% CI, 0.654–0.852) with a sensitivity of 55.56% and a specificity of 86.67%.^15^ The combined use of PSP and CRP improved the accuracy to 0.79 (95% CI, 0.72–0.86) with a cut-off point of 290.5 ng/ml for PSP and 167.2 mg/l for CRP for the prediction of sepsis.^20^

ROC curve analysis of our data revealed the prognostic value of these biomarkers for mortality. We found PSP and pSOFA had the most prognostic value for mortality detection having the same AUC of 0.709, with 74% sensitivity and 72% specificity with a cut-off point of 54 ng/ml for PSP, and pSOFA had a sensitivity of 74% and a specificity of 62% with a cut-off point of 6.5. Copeptin AUC was 0.705 with a cut-off level of 3.63 ng/ml, 58% sensitivity, and 76% specificity. However, APOA5 had a lower AUC of 0.571 with a cut-off point of 951 ng/ml, 58% sensitivity, and 53% specificity.

In a study of the prognostic value of PSP and CRP for 28-day mortality in children, ROC curve analysis revealed a PSP AUC value of 0.73 (95% CI, 0.67–0.79), with a sensitivity of 79.7% and a specificity of 57.7% with a cut-off point of 256 ng/ml. For CRP, the AUC value was 0.76 (95% CI, 0.70–0.82), with a sensitivity of 72.1%, a specificity of 68.1%, and a cut-off point of 47 mg/l.^23^ ROC curve analysis for mortality predictors showed an AUC value of 0.735 (95% CI, 0.642–0.827) for copeptin concentration.^17^

The pSOFA score provided excellent discrimination of in-hospital mortality in critically ill children with sepsis (AUC, 0.92; 95% CI, 0.91–0.94) and its performance was better than the other organ dysfunction scores and similar to PRISM III (AUC 0.88; 95% CI, 0.86–0.91), with a mortality rate of 12.1% in children with sepsis, and a mortality rate of 32.3% in children with septic shock.^30,31^ A previous study demonstrated the ability of the pSOFA score to predict mortality in PICU patients with sepsis with a sensitivity of 83% (95% CI, 76–88%) and specificity of 72% (95% CI, 60–81%).

APOA5 has also demonstrated promise in providing accurate PICU mortality prognoses, as in a previous study, the AUC value of APOA5 levels was 0.789 (95% CI, 0.593–0.984) with a sensitivity of 75% and a specificity of 83.6% at a cut-off value of 822 ng/ml.^15^

In comparison, the PRISM III and PIM scores, as well-known prognostic discriminators, had lower AUC values (0.611 and 0.573, respectively) for mortality prediction and relatively lower sensitivity than serum PSP and copeptin. These results support the use of serum PSP and copeptin as prognostic sepsis biomarkers in children. Another study examining the PRISM III score AUC to predict PICU mortality was 0.741 (95% CI, 0.552–0.930). At the cut-off value of 13, with a sensitivity of 54.6% and a specificity of 85.6%.^15^

## Conclusion

APOA5, copeptin, and PSP are acute-phase proteins with diagnostic value in evaluating critically ill pediatric patients with sepsis and detecting sepsis severity. PSP and copeptin serum concentrations were positively associated with mortality, and they had the power to discriminate non-survivors from survivors. APOA5 serum concentrations were negatively associated with mortality but were less powerful than the other biomarkers in discriminating between survivors and non-survivors. One limitation of this research is that serum levels of APOA5, copeptin, and PSP were measured only once, and changes in their serum concentrations in response to medication and other factors could not be observed.

## Data Availability

The datasets generated during and/or analyzed during the current study are available from the corresponding author on reasonable request.
